# Differentiation of Antibodies against Selected Simbu Serogroup Viruses by a Glycoprotein Gc-Based Triplex ELISA

**DOI:** 10.3390/vetsci8010012

**Published:** 2021-01-18

**Authors:** Kerstin Wernike, Andrea Aebischer, Franziska Sick, Kevin P. Szillat, Martin Beer

**Affiliations:** 1 Institute of Diagnostic Virology, Friedrich-Loeffler-Institut, 17493 Greifswald-Insel Riems, Germany; franziska.sick@fli.de (F.S.); kevin.szillat@fli.de (K.P.S.); 2 Department of Experimental Animal Facilities and Biorisk Management, Friedrich-Loeffler-Institut, 17493 Greifswald-Insel Riems, Germany; andrea.aebischer@fli.de

**Keywords:** *Peribunyaviridae*, orthobunyavirus, Schmallenberg virus, Shuni virus, Akabane virus, serology, ruminants, cattle, sheep, goat

## Abstract

The Simbu serogroup of orthobunyaviruses includes several pathogens of veterinary importance, among them Schmallenberg virus (SBV), Akabane virus (AKAV) and Shuni virus (SHUV). They infect predominantly ruminants and induce severe congenital malformation. In adult animals, the intra vitam diagnostics by direct virus detection is limited to only a few days due to a short-lived viremia. For surveillance purposes the testing for specific antibodies is a superior approach. However, the serological differentiation is hampered by a considerable extent of cross-reactivity, as viruses were assigned into this serogroup based on antigenic relatedness. Here, we established a glycoprotein Gc-based triplex enzyme-linked immunosorbent assay (ELISA) for the detection and differentiation of antibodies against SBV, AKAV, and SHUV. A total of 477 negative samples of various ruminant species, 238 samples positive for SBV-antibodies, 36 positive for AKAV-antibodies and 53 SHUV antibody-positive samples were tested in comparison to neutralization tests. For the newly developed ELISA, overall diagnostic specificities of 84.56%, 94.68% and 89.39% and sensitivities of 89.08%, 69.44% and 84.91% were calculated for SBV, AKAV and SHUV, respectively, with only slight effects of serological cross-reactivity on the diagnostic specificity. Thus, this test system could be used for serological screening in suspected populations or as additional tool during outbreak investigations.

## 1. Introduction

Viruses of the Simbu serogroup, which belongs to the family *Peribunyaviridae*, genus *Orthobunyavirus*, are distributed worldwide and include several pathogens of veterinary importance [1,2], e.g., members of the virus species *Akabane orthobunyavirus*, *Schmallenberg orthobunyavirus* or *Shuni orthobunyavirus* [3]. Historically, viruses were assigned into this serogroup solely based on antigenic relation determined by plaque reduction neutralization, hemagglutination inhibition, complement fixation or radial immunodiffusion tests [4,5]. More recently, the classification was additionally based on the comparative analyses of nucleic acid and protein sequences [2]. The genome of simbuviruses consists of three segments of single-stranded RNA, of which the small (S) genomic segment, that encodes for the nucleocapsid protein N and the non-structural protein NSs, is the most conserved one. The large (L) segment encodes for the RNA-dependent RNA polymerase and the medium (M) segment for the glycoproteins Gn and Gc and the non-structural protein NSm [6,7]. The orthobunyaviral glycoproteins, which form spikes on the surface of the virus particle, are integral transmembrane proteins and they are important for viral attachment, membrane fusion and the induction of the host’s immune response [7,8,9,10]. The N-terminal variable part of the Gc-protein (Gc head (GcH)) is highly immunogenic and the major target of neutralizing antibodies [11,12].

Simbuviruses infect predominantly ruminants and persist in nature by alternately infecting their insect vectors (*Culicoides* biting midges) and mammalian hosts (reviewed in [1]). In enzootic regions, Simbu serogroup viruses usually establish patterns of cyclic circulation, with seasons of high virus appearance followed by periods of only sporadic detections [13,14,15,16,17,18,19], which is most likely related to the overall immunity in the mammalian host population and the abundance of the insect vector. Co‑circulation of several simbuviruses within a given area occurs frequently [2,20,21,22,23].

Infections of adult ruminants with Akabane virus (AKAV), Schmallenberg virus (SBV) or Shuni virus (SHUV) lead to a short-lived viremia of a few days and are either asymptomatic or induce mild clinical signs such as fever, diarrhea and decreased milk yield [24,25,26,27]. However, some strains of AKAV and SHUV can also occasionally cause encephalitis [25,26,28]. Nevertheless, the most prominent clinical signs appear when naïve dams are infected during a critical phase of gestation, which may lead to abortion, premature or stillbirth or severe congenital deformation summarized under the term arthrogryposis-hydranencephaly syndrome [24,26,29,30]. Besides the main hosts, i.e., domestic and various captive and wild ruminants, AKAV infections of pigs [31], SHUV infection of horses [32] and anti-SBV antibodies in wild boar [33,34] and a few SBV-positive dogs [35,36] have been described. The epidemiological relevance, however, is not known.

In ruminants of all age groups, antibodies against simbuviruses are induced between one and three weeks after infection [25,37,38,39,40], and immunity acquired due to an earlier infection or vaccination protects from re-infection [37,41,42]. In cases of in utero infections of fetuses that are already immunocompetent (from about 90 days of gestation onwards in cattle), antibodies can be detected in the blood of the newborn before the intake of the colostrum of its mother [40,43,44]. Test systems commonly used to measure the humoral immune response include microneutralization and indirect immunofluorescence tests as well as (commercial) enzyme-linked immunosorbent assays (ELISAs). As viruses are assigned into this group based on antigenic relatedness [4], considerable serological cross-reactivity occurs between different simbuviruses [45], especially for tests that rely on the viral N-protein, such as commercial ELISAs or complement fixation tests [4,21,46,47]. The N-protein is widely used for serological diagnostics, as it elicits a strong humoral immune response [42,48,49] and anti‑N antibodies are highly abundant in infected animals, however, they do not have neutralizing activity [50]. In contrast, antibodies directed against the glycoproteins, specifically Gc, efficiently neutralize the corresponding simbuvirus [11,12,50]. In addition, the M-segment, which encodes the glycoproteins, is considered to be the most variable genomic segment of orthobunyaviruses [45], presumably leading to a lower extent of cross-reactivity compared to the N-protein. Indeed, serum neutralization tests (SNTs), which detect neutralizing antibodies directed against the glycoproteins, are more specific for a given virus species [46]. However, these test systems require the handling of the respective virus, are time-consuming and labor-intensive. In contrast, ELISAs can be applied under less stringent biosafety conditions and enable high-throughput testing of clinical specimens. Hence, we established a test system for the differentiation of antibodies against the Simbu serogroup viruses SBV, AKAV, and SHUV based on the viral Gc-proteins and a triplex ELISA platform.

## 2. Materials and Methods

### 2.1. Blood Samples

A total of 477 serum or plasma samples negative for antibodies against Simbu serogroup viruses taken from cattle, sheep, goat or wildlife and zoo animals (red deer, doe deer, fallow deer, buffalo and alpaca) were included (Table 1). They were collected prior to the first detection of a simbuvirus in Germany, i.e., before 2011, or represented pre-infection sera of SBV, AKAV or SHUV cattle or sheep trials. In addition, 238 samples positive for SBV-antibodies, 36 sera displaying anti-AKAV antibodies and 53 SHUV-positive samples were included (Table 1). These specimens represented either routine diagnostic submission to the Friedrich-Loeffler-Institut, Germany, to the Kimron Veterinary Institute, Israel, or originated from cattle and sheep experimentally infected with SBV [51,52], cattle infected with SHUV [25] or cattle vaccinated against AKAV, the Simbu serogroup member Aino virus (AINOV) and the likewise teratogenic Chuzan virus [53]. The sera of experimentally infected animals were collected between 14 and 28 days (SBV) or 7 and 21 days (SHUV) after infection. The Israeli field sera were pre-selected based on a positive result in a commercial SBV ELISA (ID Screen Schmallenberg virus Competition Multispecies, IDvet, Grabels, France) that detects antibodies against several Simbu serogroup viruses [46,47]. The German field sera were pre-selected based on the result of either the ID Screen Schmallenberg virus Competition Multispecies or ID Screen Schmallenberg virus Indirect Multispecies ELISA (both IDvet, Grabels, France).

The status of each sample was determined by microneutralization tests against SBV, AKAV and SHUV performed as described previously [25,37,53]. When neutralizing antibodies against several simbuviruses could be detected, the sera were evaluated positive for the virus that displayed the highest titer. If the titer difference between two viruses was less than 4-fold, the serum was assessed positive for both.

### 2.2. Production of Recombinant Proteins

#### 2.2.1. Cloning

The sequences of Gc head domains of SBV (amino acids (aa) 465–702), AKAV (aa 465–701) and SHUV (aa 465–704) were amplified from codon-optimized synthetic genes (Thermo Fisher Scientific, Darmstadt, Germany) based on GenBank entries CCF55030 (SBV), BAV17033.1 (AKAV) and KF153117 (SHUV), respectively, and cloned in the pMT *Drosophila* S2 expression vector (Thermo Fisher Scientific) in frame with an N-terminal BiP secretion signal. C-terminally, a double (SBV, AKAV) or a single (SHUV) Strep-Tag was added. All constructs were verified by Sanger sequencing. Primer sequences are available upon request.

#### 2.2.2. Expression and Purification in Drosophila S2 Cells

*Drosophila* S2 cells (R69007) were purchased from Thermo Fisher Scientific and grown in Insect-Xpress medium (Lonza, Basel, Switzerland) at 28 °C. For protein expression, adherent cultures were transfected with the respective pMT/BiP expression plasmids and pCoBlast (Invitrogen, Karlsruhe, Germany) in a ratio of 20:1 using Effectene Transfection Reagent (#301425, Qiagen, Hilden, Germany) according to the instructions of the manufacturer. Stable polyclonal cell lines were subsequently selected by addition of 30 µg/mL Blasticidin and expanded to suspension cultures of 300 mL and grown at 28 °C, 80 rpm. After 5 days, the cultures were topped to 700 mL and protein expression was induced with a final concentration of 2.5 µM CdCl_2_. Supernatants were harvested 7 days after induction by centrifugation and were subsequently concentrated to about 50 mL using a Vivaflow 200 device (5000 MWCO PES; Sartorius, Göttingen, Germany). Biotin was blocked by addition of BioLock (IBA Lifesciences, Göttingen, Germany) as recommended. The concentrated supernatant was purified using Streptactin-Superflow high capacity slurry (IBA Lifesciences) according to the manufacturer’s protocol. All protein-containing eluates were pooled, aliquoted and stored at –80 °C until further use.

#### 2.2.3. SDS-PAGE and Western Blot

Sodium dodecyl sulfate polyacrylamide gel electrophoresis (SDS-PAGE) was performed using a Mini-PROTEAN^®^Tetra System (Bio-Rad, Feldkirchen, Germany). InstantBlue (expedeon, Heidelberg, Germany) was applied for Coomassie stainings. For Western blot analysis a TRANS-BLOT ^®^ SD semi-dry transfer cell (Bio-Rad) was used. Stainings were performed with an Anti-StrepTag specific antibody (StrepMAB-Classic-HRP, IBA Lifesciences). Images were acquired with a ChemiDoc Imaging System (Bio-Rad).

### 2.3. ELISA Procedure

Medium-binding ELISA plates (Greiner Bio-One GmbH, Leipzig, Germany) were coated with 100 ng/well of either the SBV, SHUV or AKAV antigen overnight at 4 °C in a Tris buffer (0.02 mol Tris and 0.15 mol NaCl ad. 1 L H_2_O, pH 7.6). The plates were subsequently washed three times using Tris-buffered saline with Tween (TBST) and blocked for 1 h at 37 °C using 5 % skimmed milk in phosphate-buffered saline (PBS). Fifty µL of the sera (pre-diluted 1/100 in TBST) were incubated on the coated wells for 1 h at room temperature followed by three washing steps using TBST. The reactivity was shown by adding 50 µL of a multi-species conjugate (SBVMILK; IDvet, Grabels, France) diluted 1/80. After an incubation period of 1 h at room temperature, the plates were washed again and 100 µL o-phenylenediamine dihydrochloride (OPD) substrate was added. Subsequent to an incubation period of 20 min at room temperature in the dark, the reaction was stopped using 50 µL 4M H_2_SO_4_. The ELISA readings were taken at a wavelength of 492 nm on a Tecan Spectra Mini instrument (Tecan Group Ltd., Männedorf, Switzerland). The results were expressed as the percentage of the sample adsorbance (= optical density (OD)) relative to the positive control OD (S/P*100).

### 2.4. Data Analyses and Cut-off Determination

To determine the cut-off values and the diagnostic sensitivities and specificities of the final ELISA protocol, the sera mentioned above were tested by the triple ELISA, and receiver operating characteristic (ROC) analyses were performed using GraphPad Prism version 8.0 for Windows (GraphPad Software, San Diego, CA, USA). To evaluate the influence of cross-reactivity with antibodies against other Simbu serogroup viruses, the ROC analyses was performed in two distinct settings. In the first set-up, only sera negative against all three simbuviruses were included as negative samples, while in the second approach negative samples additionally include sera negative against the given antigen, but positive for another Simbu serogroup virus.

### 2.5. Repeatability and Reproducibility

For evaluation of the intra-assay reproducibility, a negative cattle and a negative sheep serum as well as cattle sera positive for anti-SBV [51], anti-AKAV [53] or anti-SHUV [25] antibodies were tested in five replicates each. The inter-assay repeatability was determined with the identical samples and replicate number on five days. Mean values and standard deviations of the 25 replicates were calculated using GraphPad Prism version 8.0 for Windows (GraphPad Software, USA). In every approach, two positive controls per virus and two negative controls were included, resulting in a total of 10 replicates per control.

## 3. Results

### 3.1. Expression and Purification of Proteins

**Figure 1:**
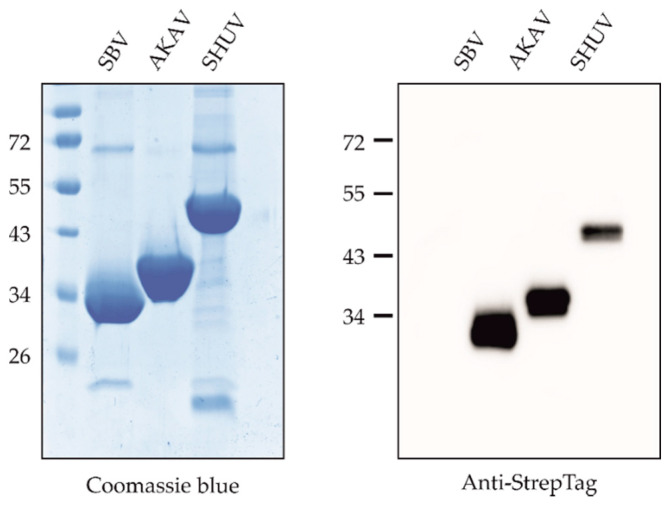
SDS-PAGE and Western blot analyses of recombinant proteins used for plate coating. The recombinant proteins were separated by reducing SDS-PAGE and analyzed by Coomassie staining (left panel) or Western blot (right panel) using an anti-StrepTag specific monoclonal antibody. Full-length blots are presented in the Appendix A (Figure A1).

The Gc head domains of SBV (aa 465–702), AKAV (aa 465–701) and SHUV (aa 465–704) were expressed to high yields in *Drosophila* S2 cells and Streptactin-purified without further downstream processing. Integrity and purity were verified by SDS-PAGE and Western blot (Figure 1). It has been shown before, that the orthobunyavirus Gc head domain represents the major target of neutralizing antibodies produced upon virus infection [11], and the recombinant SBV, AKAV and SHUV ELISA antigens were designed and produced based on this previously defined molecular architecture of the Gc spike protein. Their antigenicity was verified by ELISA using the antibody-positive and -negative sera as shown below.

### 3.2. Cut-off Determination and Diagnostic Characteristics

**Figure 2:**
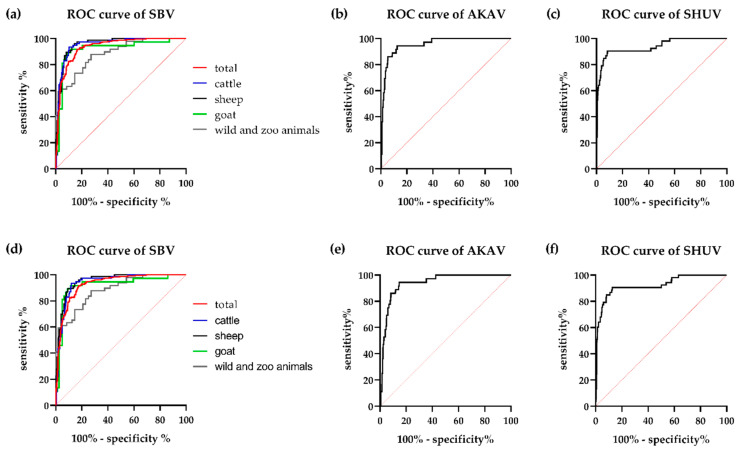
Receiver operating characteristic (ROC) analyses of the triplex SBV–AKAV–SHUV ELISA using 477 negative sera and 238 SBV antibody-positive sera, 36 sera displaying antibodies against AKAV and 53 SHUV seropositive samples. For the SBV assay (**a**,**d**), the ROC curves are shown separately for cattle (blue), sheep (black), goat (green) and wild and zoo animals (grey), the combined ROC curve that includes all species is depicted in red. The ROC analyses were performed using two different set-ups, for the first (**a**–**c**) only sera negative against all three simbuviruses were included as negative samples, while in the second approach (**d**–**f**) negative samples additionally include sera negative against the given antigen, but positive towards another Simbu serogroup virus. SBV, Schmallenberg virus; AKAV, Akabane virus; SHUV, Shuni virus.

In order to evaluate the sensitivity and specificity of the triplex SBV-AKAV-SHUV-ELISA and to establish a threshold for positivity, the above-mentioned antibody-negative and -positive sera of multiple ruminant species were tested. ROC curve analyses indicated that the individual ELISAs have a good diagnostic accuracy with only a very limited negative effect of cross-reactive antibodies (Figure 2). The areas under the curve (AUC) that were calculated for two selected test set-ups are given in Table 2. Based on the ROC curves (Figure 2), a cut-off of <50% for negativity and ≥50 for positivity was set for cattle, sheep and wild and zoo animals and of <80% and ≥80%, respectively, for goat samples.

Using these cut-off values, overall diagnostic specificities of 84.56%, 94.68% and 89.39% and sensitivities of 89.08%, 69.44% and 84.91% were achieved for SBV, AKAV and SHUV, respectively. Taking in account differences between set-ups 1 and 2, only a limited impact of cross-reacting antibodies could be observed (Table 2). When comparing the diagnostic characteristics of the SBV test separately for each animal species included in this study, a higher sensitivity was observed when testing domestic ruminants (cattle, goat, sheep) in comparison to wild and zoo animals (Table 2).

**Figure 3:**
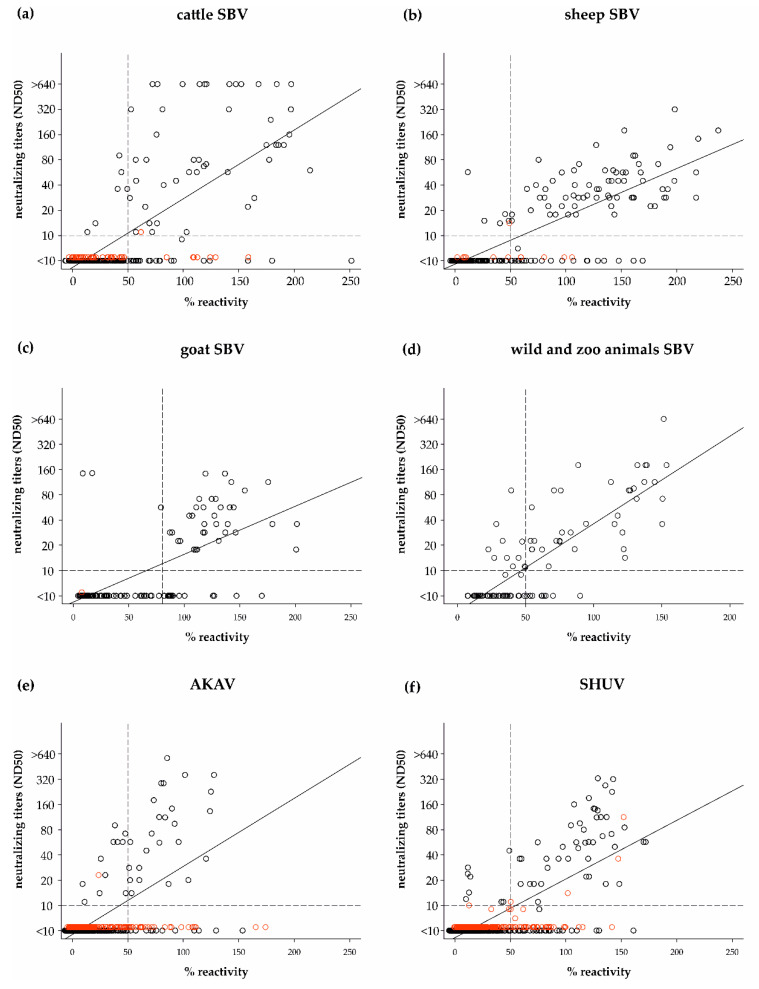
Correlation of the results of serum neutralization tests (SNT) against SBV (**a**–**d**), AKAV (**e**) and SHUV (**f**) and the ELISA based on the respective antigen. Red dots represent samples that tested positive by serum neutralization tests (SNT) against a different simbuvirus. The cut-off value for the SNT is indicated by a horizontal dashed line, while the cut-off values of the ELISA are shown by vertical dashed lines.

All sera were additionally tested by the gold-standard test SNT against all three viruses and the measured neutralizing titers were compared to the ELISA values. Overall, the proportion of false-negative ELISA results was higher for samples with low neutralizing titers (<1/80), an effect most prominent for previously AKAV-infected individuals or SBV-antibody-positive wildlife and zoo animals (Figure 3).

### 3.3. Repeatability and Reproducibility

**Figure 4:**
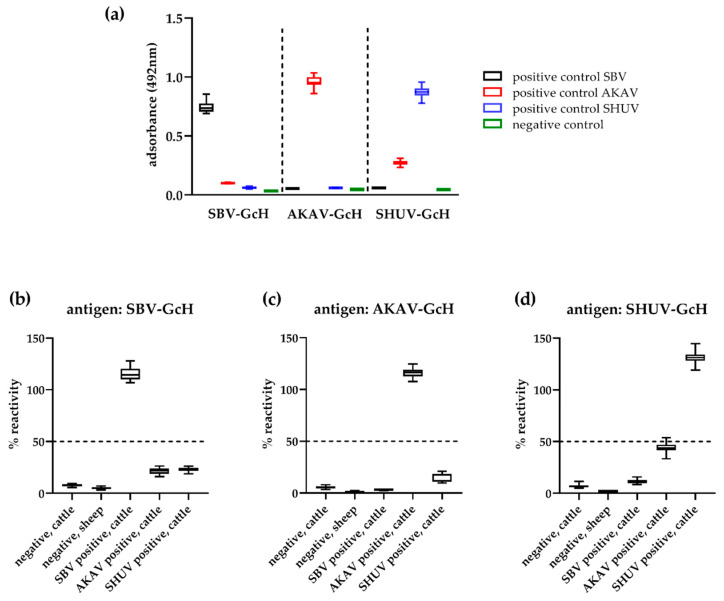
Reproducibility and repeatability of the SBV-AKAV-SHUV ELISA. A negative cattle and a negative sheep serum as well as sera antibody-positive towards SBV, AKAV or SHUV were tested against the SBV (**b**), AKAV (**c**) and SHUV (**d**) antigens in five replicates each in five independent approaches. The boxplots represent the results of all 25 replicates. The cut-off values are indicated by horizontal dashed lines. (**a**) Adsorbance values of the negative and positive controls that were included in every approach, the boxplots represent the results of all 10 replicates.

The repeatability and reproducibility were assessed using five replicates each of five sera on five independent ELISA plates. The included control samples reacted as expected in every approach and with low standard deviations (SD) (Figure 4a). The SBV control resulted in an adsorbance value of 0.75 ± 0.05 against the SBV antigen, while only values of 0.05 ± 0.00 and 0.06 ± 0.00 were measured when testing this sample against the AKAV and SHUV antigens, respectively. The AKAV control sample, which originated from an animal vaccinated against AKAV as well as AINOV and Chuzan virus [53], resulted in adsorbance values of 0.96 ± 0.05, 0.10 ± 0.00 and 0.27 ± 0.02 when tested against the AKAV, SBV and SHUV antigens, respectively. The values measured for the SHUV control samples were 0.87 ± 0.05 against the SHUV antigen and 0.06 ± 0.01 and 0.06 ± 0.00 against the SBV and AKAV proteins (Figure 4a).

The results of the clinical specimens to be analyzed resulted likewise in low variations between the individual approaches. The negative cattle and sheep sera tested correctly negative in every case (Figure 4b–d). Mean OD values and standard deviations of 7.80% ± 1.18 (cattle) and 5.10% ± 1.10 (sheep) were calculated for these samples when tested against the SBV antigen, 5.58% ± 1.24 and 1.34% ± 0.53 against AKAV and 6.89% ± 1.26 and 2.02% ± 0.29 against SHUV-GcH. The SBV seropositive sample reacted correctly positive against SBV (115.12% ± 5.48) in every approach and negative against AKAV (3.14% ± 0.34) and SHUV (11.22% ± 1.93), while the SHUV antibody-positive sample reacted positive against SHUV (131.25% ± 5.73) and negative against SBV (23.22% ± 1.85) and AKAV (15.35% ± 3.73) in every case. The cattle sample that contains antibodies against AKAV reacted positive against this antigen (116.30% ± 4.19%) and negative against SBV (21.53% ± 2.76) (Figure 4b,c), however, against the SHUV antigen elevated values close to the cut-off for positivity were obtained (mean 44.10%, SD 4.39, max. 53.86%) (Figure 4d).

## 4. Discussion

During the last decades, numerous new members of the large and diverse family *Peribunyaviridae* were discovered and known bunyaviruses, among them several Simbu serogroup viruses, were found in previously unaffected regions or in areas with unknown infection status [7,27,30,54,55,56]. However, in some cases, predominantly in large-scale screenings, only antibodies against simbuviruses in general were detected using broad-reactive serological test systems, making it difficult to pinpoint a specific virus [47,57,58]. In those screenings conducted at a given time point, the direct virus detection and subsequent identification by specific RT-PCRs or sequence analyses is hampered by the very short viremia of only a few days [25,26,27]. In contrast, antibodies against simbuviruses are detectable in most animals for several years after infection, if not even lifelong [17,59,60,61,62], albeit one should keep in mind that acute infections cannot be diagnosed by serological methods. For serological differentiation of antibodies against the different viruses, SNTs may be used [21,47]. However, these tests are time-consuming and require the storage and handling of the respective viruses under adequate biosecurity conditions. In contrast, discriminatory ELISA tests would be easier to perform, can be easily standardized and can potentially be performed by untrained personal. Therefore, we propose to perform (large-scale) screenings by broadly-reacting commercial ELISAs in order to detect the vast majority of bunyavirus-specific antibodies. In a second step, a virus-specific ELISA should be applied for further antibody-differentiation. Finally, questionable results would need to be verified by SNT. In order to test the applicability of this approach, SBV, AKAV and SHUV were selected in this study as model orthobunyaviruses to design an indirect triplex ELISA system allowing differentiation of antibodies raised against these viruses. SBV was chosen for this proof-of-principle study since it represents by now one of the best characterized orthobunyaviruses and, more importantly, SBV serological tests are widely used to screen for antibodies against simbuviruses [21,47], in some cases without further differentiation or confirmation by more specific tests [63,64]. AKAV and SHUV were added as they are widespread [1] and occur in the same geographical regions, including the Middle East [20] and the African continent. Furthermore, SHUV-specific ELISA systems are not yet described. For the selection of ELISA antigens, we focused on the N-terminal domain of the orthobunyavirus Gc-protein, since it shows a higher sequence variability between different viruses of a given group, while the C-terminal part is well conserved. Furthermore, the Gc ectodomain in general is highly immunogenic and represents the major target of neutralizing antibodies [11,12,50]. Hence, based on these previous findings, the Gc head domain was considered to be the most promising antigen for a discriminatory ELISA approach. It was shown before, that the complete Gc head-stalk domain represents a superior antigen in comparison to the head domain only [11]. However, we chose the isolated SBV and AKAV head domains since their antigenicity and immunogenicity have been confirmed before both, in vitro and in vivo [12,49,65]. Based on structural data [11], we assumed an equivalent performance also for SHUV, but no experimental data was available for verification. To ensure an optimal antigenicity, we therefore decided to use the complete SHUV Gc head-stalk domain. Its suitability for the design of antibody detection systems could be demonstrated in this study.

With regard to protein expression, one should take into account that a functional Gc glycoprotein requires correct post-translational modifications to ensure a proper folding [12,65], in contrast to the N-protein, which can be easily produced in a bacterial expression system. Thus, the recombinant glycoproteins have to be produced in a suitable expression host. Here, we used insect *Drosophila* S2 cells, which provide an eukaryotic environment as well as the majority of post-translational modifications found in mammalian cells [66]. In previous studies, the S2-expressed SBV Gc head domain was fully functional and showed an equal or even better performance than the same antigen expressed in mammalian HEK293T cells [11,49]. Furthermore, the S2 system can be easily up-scaled for high yield production of secreted proteins at relatively low costs. This represents a further benefit with regard to production under resource-limited settings. All the S2-expressed recombinant proteins reacted readily with sera from infected or immunized animals and can therefore be considered as fully functional. Moreover, only a limited extend of cross-reactivity was observed between the SBV-, AKAV- and SHUV antigens clearly out-performing the specificity of N-based serological assays [21,46,47]. However, it cannot be fully excluded that Gc-specific antibodies against more closely related members of this serogroup, e.g., from the identical virus species, would result in unspecific reactions. Thus, this needs to be investigated in future studies. Nonetheless, the AKAV positive control serum and the AKAV serum used to determine the reproducibly did react in the SHUV-specific system only to a limited extend and remained under the cut-off for positivity (Figure 4), although both of these sera were obtained from animals vaccinated with a trivalent vaccine also containing AINOV, which is more closely related to SHUV [45].

In terms of sensitivity, satisfactory results were achieved for SBV and SHUV, when specimens from domestic ruminants were tested. In contrast, lower sensitivities were observed for samples collected from wild or zoo animals or in the AKAV test. However, among the samples used for validation a relatively high percentage of specimens with low titers (<1/80) as measured in the gold standard test SNT were present in the sample subsets positive for AKAV antibodies or among the wildlife samples. Hence, the lower sensitivities might be related to some extent also to the highly demanding sample panel. In addition, test sensitivity might be influenced by sampling time-points, as it was previously described that antibody titers are decreasing over time after infection in some animals [17]. In terms of age, also the group of young animals could be problematic, as maternally-derived antibodies transferred via colostrum from the dam to its newborn decay within a few months [59,60]. Thus, dependent on the age of the offspring, only very small amounts of these antibodies might still be present in the bloodstream. However, an influence of the sampling time-point on the performance of the ELISAs could not be further evaluated in this study, due to the missing information on the age and infection status of animals sampled for routine diagnostics. Nevertheless, a good overall correlation between the ELISAs and the corresponding SNTs could be observed, except for some samples with very low antibody-titers. Thus, we recommend an initial screening with highly sensitive N-based ELISAs to detect also small amounts of antibodies. Since the N-protein is produced at high levels in infected cells, N-specific antibodies occur abundantly and very early after infection [37]. In contrast, Gc-specific neutralizing antibodies can be detected reliably only from two to three weeks after infection onwards [37]. We therefore suggest using the Gc-ELISA in a second step primarily to further differentiate virus-specific antibodies.

In this study, we present a strategy to rapidly design ELISA tests allowing discrimination of antibodies against different orthobunyaviruses. Based on the conserved molecular architecture of their spike protein we suppose that similar assays can easily be adopted for additional bunyaviruses. Such tests are valuable tools to assess occurrence and distribution of specific viruses in regions where several simbuviruses (or other bunyaviruses) are supposed to circulate and, as a consequence, serological tests are needed to differentiate those pathogens.

## 5. Conclusions

A novel, highly specific triplex ELISA system for the differentiation of antibodies against SBV, AKAV and SHUV was developed. This assay could serve as a basis for the fast establishment of ELISAs against various further orthobunyaviruses in the future, as the antigenicity of three distinct proteins that were produced according to a previously defined general molecular architecture of the complete genus was demonstrated. Such ELISAs may be used for high-throughput serological screenings in suspected populations or as additional tools during outbreak investigations in regions in which several orthobunyaviruses circulate.

## Figures and Tables

**Table 1:** Numbers and serological status towards simbuviruses of samples included in this study. Wild and zoo animals comprise red deer, roe deer, fallow deer, buffalo and alpaca.

Animal Species	No. Negative	No. Positive against SBV	No. Positive against AKAV	No. Positive against SHUV
cattle	192	76	30	42
sheep	159	76	5	11
goat	78	37	1	0
wild and zoo animals	48	49	0	0
total	477	238	36	53

**Table 2:** Diagnostic characteristics of the triplex SBV-AKAV-SHUV ELISA. The first row per antigen and, if applicable, animal species includes values calculated for samples positive against the given antigen and negative against all simbuviruses. In the second row, negative samples additionally include sera negative against the given antigen, but positive for another Simbu serogroup virus. AUC: area under the curve, CI: 95% confidence interval.

Antigen	Animal Species	AUC	Sensitivity	Specificity
SBV	cattle-1	0.9519 (CI: 0.9259 to 0.9779)	92.11% (CI: 83.60% to 97.05%)	89.58% (CI: 84.37% to 93.52%)
	cattle-2	0.9418 (CI: 0.9149 to 0.9686)	92.11% (CI: 83.60% to 97.05%)	87.98% (CI: 83.10% to 91.86%)
	sheep-1	0.9566 (CI: 0.9326 to 0.9806)	93.42% (CI: 85.31% to 97.83%)	86.79% (CI: 80.52% to 91.63%)
	sheep-2	0.9532 (CI: 0.9289 to 0.9775)	93.42% (CI: 85.31% to 97.83%)	84.80% (CI: 78.52% to 89.82%)
	goat-1	0.9186 (CI: 0.8553 to 0.9818)	94.59% (CI: 81.81% to 99.34%)	76.92% (CI: 66.00% to 85.71%)
	goat-2	0.9196 (CI: 0.8571 to 0.9821)	94.59% (CI: 81.81% to 99.34%)	77.22% (CI: 66.40% to 85.90%)
	wild/zoo animals-1	0.8835 (CI: 0.8199 to 0.9471)	73.47% (CI: 58.92% to 85.05%)	79.17% (CI: 65.01% to 89.53%)
	wild/zoo animals-2	0.8835 (CI: 0.8199 to 0.9471)	73.47% (CI: 58.92% to 85.05%)	79.17% (CI: 65.01% to 89.53%)
	overall-1	0.9339 (CI: 0.9159 to 0.9520)	89.08% (CI: 84.40% to 92.74%)	85.53% (CI: 82.05% to 88.57%)
	overall-2	0.9292 (CI: 0.9110 to 0.9474)	89.08% (CI: 84.40% to 92.74%)	84.56% (CI: 81.20% to 87.53%)
AKAV	overall-1	0.9536 (CI: 0.9247 to 0.9825)	69.44% (CI: 51.89% to 83.65%)	97.06% (CI: 95.12% to 98.39%)
	overall-2	0.9386 (CI: 0.9079 to 0.9692)	69.44% (CI: 51.89% to 83.65%)	94.68% (CI: 92.80% to 96.19%)
SHUV	overall-1	0.9384 (CI: 0.8997 to 0.9771)	84.91% (CI: 72.41% to 93.25%)	93.29% (CI: 90.66% to 95.37%)
	overall-2	0.9234 (CI: 0.8792 to 0.9676)	84.91% (CI: 72.41% to 93.25%)	89.39% (CI: 86.89% to 91.55%)

**Figure A1:**
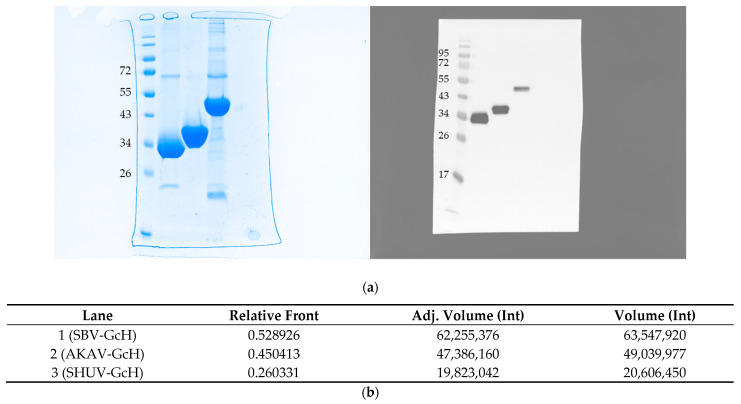
(**a**) Full-length blots of SDS-PAGE and Western blot analyses of the recombinant proteins shown in Figure 1. (**b**) The band intensities (Western blot) of the SBV, AKAV and SHUV proteins were analyzed using the Image Lab software (Bio-Rad, Feldkirchen, Germany).

## Data Availability

The data presented in this study are available in the article and Appendix A.
